# Single Stranded DNA Viruses Associated with Capybara Faeces Sampled in Brazil

**DOI:** 10.3390/v11080710

**Published:** 2019-08-02

**Authors:** Rafaela S. Fontenele, Cristiano Lacorte, Natalia S. Lamas, Kara Schmidlin, Arvind Varsani, Simone G. Ribeiro

**Affiliations:** 1 The Biodesign Center for Fundamental and Applied Microbiomics, Center for Evolution and Medicine School of Life Sciences, Arizona State University, Tempe, AZ 85287, USA; 2 Embrapa Recursos Genéticos e Biotecnologia, Brasília, DF 70770-017, Brazil; 3 Structural Biology Research Unit, Department of Clinical Laboratory Sciences, University of Cape Town, Observatory, Cape Town 7925, South Africa

**Keywords:** *Hydrochoerus hydrochaeris*, CRESS DNA virus, *Circoviridae*, *Genomoviridae*, *Microviridae*, *Smacoviridae*

## Abstract

Capybaras (*Hydrochoerus hydrochaeris*), the world’s largest rodents, are distributed throughout South America. These wild herbivores are commonly found near water bodies and are well adapted to rural and urban areas. There is limited information on the viruses circulating through capybaras. This study aimed to expand the knowledge on the viral diversity associated with capybaras by sampling their faeces. Using a viral metagenomics approach, we identified diverse single-stranded DNA viruses in the capybara faeces sampled in the Distrito Federal, Brazil. A total of 148 complete genomes of viruses in the *Microviridae* family were identified. In addition, 14 genomoviruses (family *Genomoviridae*), a novel cyclovirus (family *Circoviridae*), and a smacovirus (family *Smacoviridae*) were identified. Also, 37 diverse viruses that cannot be assigned to known families and more broadly referred to as unclassified circular replication associated protein encoding single-stranded (CRESS) DNA viruses were identified. This study provides a snapshot of the viral diversity associated with capybaras that may be infectious to these animals or associated with their microbiota or diet.

## 1. Introduction

Capybaras (*Hydrochoerus hydrochaeris*, Linnaeus 1766) are the world’s largest rodent and have a wide distribution throughout South America. They are herbivores with a generalist diet, and their semi-aquatic habit requires the presence of water bodies such as lakes, rivers, and lagoons [1]. Capybaras are wild animals but also well adapted to human-modified landscapes [2,3]. Due to their high reproductive rates, capybaras can expand their population rapidly, especially in areas with few predators such as urban environments and agricultural farms [4,5]. The proximity of capybara habitats to humans and domestic animals in urban areas can facilitate the zoonotic spread of pathogens such as *Rickettsia rickettsii* [6].

A hand full of viruses have been identified associated with capybaras. Recently, a mimivirus (family *Mimiviridae*) was identified in faecal samples of 17 capybaras collected in the Midwest and Southeast regions of Brazil [7]. Vaccinia virus (VACV) (family *Poxviridae*) has been identified in faeces of capybaras from wild and urban areas of Brazil [8] and has also been detected in serum samples by serological assays [9]. A study with experimentally infected capybaras demonstrated that VACV is able to replicate in this rodent species [10], supporting their role in the ecology of VACV [11]. Serological assays have shown the presence of Rabies virus (family *Rhabdovirus*), which is believed to be transmitted to the capybaras by blood-feeding bats [12]. Finally, following a diarrhoea outbreak from a group of capybaras in a city from São Paulo state, Brazil, a coronavirus (family *Coronavridae*) was identified based on transmission electron microscopy [13].

With the advent of high throughput sequencing (HTS) technologies, the discovery and identification of known and novel viruses have increased significantly over the last decade. Currently, there are five recognised eukaryotic-infecting circular replication associated protein encoding single-stranded (CRESS) DNA virus families: *Bacillidnaviridae* [14], *Circoviridae* [15], *Geminiviridae* [16], *Genomoviridae* [17], and *Nanoviridae* [18]. Whereas, the prokaryotic-infecting CRESS DNA viral families are *Microviridae* [19] and *Inoviridae* [20], which infect bacteria and *Pleolipoviridae* [21] and *Smacoviridae* [22]—recently identified to likely infect archaea due to the presence of CRISPR spacers identified by bioinformatics analysis [23]—that infect archaea. In addition, there are numerous novel clusters of CRESS DNA viruses that remain to be taxonomically classified. The replication associated proteins (Reps) of the eukaryotic circular Rep encoding single-stranded CRESS DNA viruses are distantly related to prokaryotic infecting ones.

With the aim to identify small DNA viruses associated with the capybara, we used a viral metagenomics approach on two faecal samples. A total of 201 viral genomes were identified; 164 span four known viral families and 37 are part of the unclassified CRESS DNA virus group.

## 2. Materials and Methods

### 2.1. Sample Collection and Processing

Two capybara faecal samples were collected in Brasilia and Planaltina, Distrito Federal, Brazil, in 2016. The fresh faecal pellet samples were picked off the ground from a grass field where wild capybaras were feeding, and were placed into a 50 mL tube. The faecal pellets were homogenised in an SM buffer (0.1 M NaCl, 50 mM Tris/HCl-pH 7.4, and 10 mM MgSO_4_) and subsequently centrifuged for 10 min at 4300 rpm. The supernatant was sequentially filtered through a 0.45 µm and 0.2 µm syringe filter and PEG-precipitated (15% *w*/*v*) overnight. The precipitated filtrate was centrifuged, and the pellet was resuspended in 1 mL of SM buffer. A measure of 200 µL of this suspension was used for viral DNA extraction using the Zymo viral purification kit (Zymo Research, Irvine, CA, USA). The extracted viral DNA was enriched for circular viral DNA using rolling circle amplification (RCA) with the Illustra TempliPhi amplification kit (GE Healthcare, Chicago, IL, USA).

### 2.2. High Throughput Sequencing and Data Analysis

The RCA products from the two faecal samples were individually sequenced on an Illumina HiSeq 2500 platform (2 × 100 paired-end library) at Macrogen Inc., Seoul, South Korea. The paired-end reads were de novo assembled using SPAdes v 3.12.0 [24], and the resulting contigs (>750 nts) were analysed by BLASTx [25] against a local RefSeq viral protein sequence database. Based on the sequence of the de novo assembled contigs that had similarity with viral sequences, abutting primers were designed to recover the full-length genome by PCR (Supplementary Table S1), with the exception of the viral full-length genome contigs (determined based on terminal redundancy of de novo assembled contigs) that belong to the family *Microviridae*. PCR was performed using HiFi HotStart DNA polymerase (KAPA Biosystems, Wilmington, MA, USA) following the manufacturer’s thermal cycling condition recommendations. The amplified genome sequences were resolved in a 0.7% agarose gel, and the expected size amplicons were gel-excised and purified using the Quick-spin PCR Product Purification Kit (iNtRON Biotechnology, Seongnam-si, South Korea). Gel purified amplicons were cloned into a pJET1.2 cloning vector (ThermoFisher Scientific, Waltham, MA, USA), and transformed into *E. coli* XL blue competent cells. The recombinant plasmids in the transformants were purified with the DNA-spin Plasmid DNA Purification kit (iNtRON Biotechnology, Seongnam-si, South Korea) and Sanger sequenced by primer walking at Macrogen Inc. (Seoul, South Korea). Sanger sequence contigs were assembled and analysed in Geneious 11.1.5 [26]. For the full genomes assembled for viruses belonging to the *Microviridae* family, the raw reads were aligned using BWA v0.7.12 [27] for coverage depth assessment. The raw read data is deposited in the SRA database (PRJNA521956). All genomes recovered in this study were deposited in the GenBank database (Accession numbers MK496679-MK496826; MK483072-MK483085; MK570163-MK570200; MK947371; Supplementary Table S1).

### 2.3. Sequence Similarity Network Analyses

A dataset (Rep_all) of the Rep proteins of the alphasatellites, circoviruses, geminiviruses, genomoviruses, nanoviruses, and smacoviruses was assembled with sequences available in GenBank. A dataset of the major capsid protein (MCP) of the microvirus sequences available in GenBank was assembled (MCP_all). Both Rep_all and MCP_all were then separately clustered with a 0.9 sequence identity cut-off using CD-HIT [28]. For the Rep analysis, a representative from each cluster and all the Reps encoded by the viruses (except microviruses) identified in this study were assembled into a dataset (Rep_90_Cap). Similarly, for the MCP, a representative from the cluster was assembled into a dataset with all the MCP sequences (MCP_90_Cap) of the microviruses identified in this study. A sequence similarity network analysis using EST-EFI [29,30] with a minimum similarity score of 60 was constructed for the Rep_90_Cap dataset and with a minimum similarity score of 200 for the MCP_90_Cap dataset. The resulting sequence similarity networks were visualised in Cytoscape V3.7.1 [31] using the organic layout.

### 2.4. Sequence Analysis

All genome-wide and protein pairwise identities were determined using SDT v1.2 [32]. BLASTp [25] analysis of the MCP of the microviruses was undertaken to determine the closest related protein sequences using the complete MCP (MCP_all) dataset.

### 2.5. Circoviruses, Genomoviruses, and Smacoviruses

The Rep amino acid sequences within the circovirus, genomovirus, and smacovirus clusters were separately aligned by MUSCLE [33]. The alignment was then used to infer a Maximum Likelihood (ML) phylogenetic tree using PhyML 3.0 [34] with rtREV+G+I (for circoviruses), rtREV+G+I+F (for genomoviruses), and rtREV+G+I+F (for smacoviruses) amino acid substitution models, inferred as best fit models using ProTest [35], and an approximate likelihood ratio test (aLRT) was used for branch support. The cyclovirus Rep ML phylogenetic tree was rooted with the representative sequences of the unclassified CRESS DNA group (Cluster 1; Figure 4). The smacovirus ML phylogenetic tree has been rooted with Rep sequences of nanoviruses. Branch support <0.8 aLRT support was collapsed using TreeGraph2 [36].

### 2.6. Unclassified CRESS DNA Viruses

Based on the network analysis, clusters with ≥4 sequences that contained the capybara CRESS DNA virus Rep sequences were phylogenetically analysed. Sequences in each cluster were aligned by MUSCLE [33]. The alignments were used to infer the ML phylogenetic tree for each cluster using PhyML 3.0 [34] with the amino acid substitution model (Clusters 1: rtREV+G+I; 2: rtREV+G+I; 3: rtREV+G+I+F; 4: rtREV+G+I+F; 5: WAG+G+I; 6: rtrev+G; 7: WAG+G; 8: rtRev+G; 9: WAG+I; 10: rtREV+G+I; and 11: WAG+I) determined as a best fit model using ProTest [35], and an approximate likelihood ratio test (aLRT) was used for branch support. All ML phylogenetic trees were midpoint rooted, and branches with <0.8 aLRT support were collapsed using TreeGraph2 [36].

### 2.7. Microviruses

Based on the network analysis, the MCP of the microviruses in the clusters with ≥5 sequences containing the MCP from the capybara associated microviruses were analysed. MCP sequences of each cluster were aligned using MUSCLE [33]. The resulting alignments were used to infer an ML phylogenetic tree using PhyML 3.0 [34] with amino acid substitution models (Clusters 1: LG+I+G+F; 2: rtRev+G+I+F; and 3: rtRev+G) determined as a best fit model using ProTest [35], with an approximate likelihood ratio test (aLRT) for branch support. All ML trees were midpoint rooted and branches with <0.8 aLRT support were collapsed using TreeGraph2 [36].

## 3. Results and Discussion

**Figure 1:**
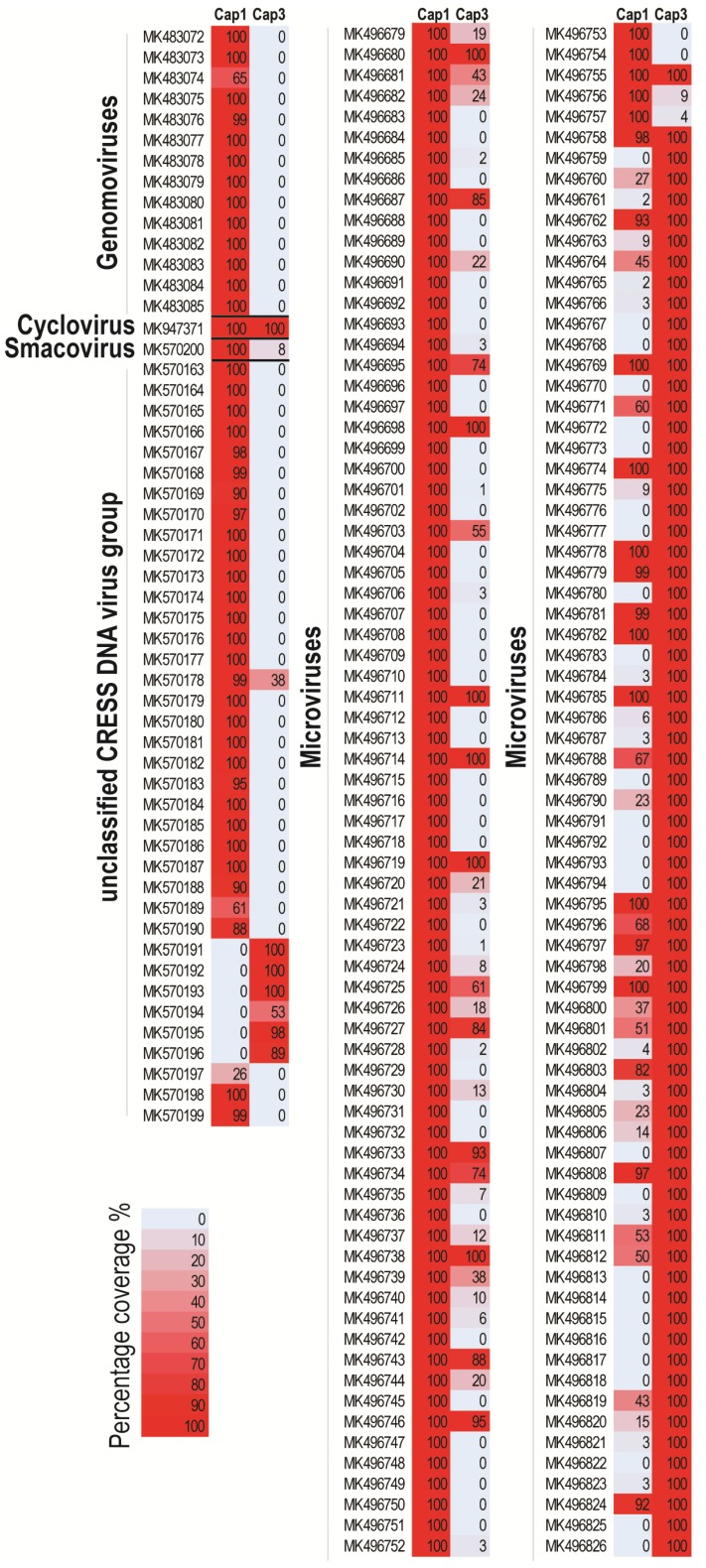
Percentage coverage of raw reads mapped from samples Cap1 and Cap3 against all genomes recovered in this study.

Two faecal samples from capybaras were collected in urban areas from Distrito Federal, Brazil and using HTS technologies, 201 CRESS DNA viruses were identified. Of these, 164 span four known viral families (*Genomoviridae*, *Circoviridae*, *Smacoviridae,* and *Microviridae*), and 37 are part of the unclassified CRESS DNA virus group (Figure 1; Supplementary Table S1). The group of CRESS DNA viruses has increased drastically in recent years due to the broad application of metagenomics approaches. However, most of the CRESS DNA viruses’ genomes remain unclassified due to their high diversity and lack of host information.

### 3.1. Classified CRESS DNA Viruses

#### 3.1.1. Genomoviruses

The family *Genomoviridae* was recently established and currently consists of nine genera [17,37]. The first isolated member of this family was the *Sclerotinia sclerotiorum* hypovirulence associated DNA virus 1 (SsHADV-1) [38]. Thus far, SsHADV-1 is the only genomovirus identified that is associated with a host, the fungus *Sclerotinia sclerotiorum*, which makes it the only fungal infecting ssDNA virus ever described [38]. The established genera within the *Genomoviridae* family are *Gemycircularvirus*, *Gemyduguivirus*, *Gemygorvirus*, *Gemykibivirus*, *Gemykolovirus*, *Gemykrogvirus*, *Gemykronzavirus*, *Gemytondvirus*, and *Gemyvongvirus* [37]. The genera demarcation has been established based on the Rep phylogenetic analysis. Within these genera, the current species demarcation threshold is 78% genome-wide pairwise identity. Genomoviruses have been isolated from a variety of environments such as fungi, plants, sediments, sewage/wastewater, insects, birds, and mammals faeces [37].

The capybara-associated genomoviruses (*n* = 14) identified in this study were all recovered from one of the capybara faecal samples (Cap1). These have been named capybara genomovirus 1–13 (CapGV1–13), with CapGV 2 having two variants, cap1_52 and cap1_64, sharing 98% genome-wide identity. These 14 genomes have classical features of genomoviruses with a conserved nonanucleotide motif at the origin of replication, and they encode a capsid protein (*cp*) in the virion sense and a replication-associated protein (*rep*) in the complementary sense (Supplementary Table S1; Supplementary Figure S1). The Rep encoded by the capybara genomoviruses all contain the rolling circle replication (RCR) endonuclease and superfamily 3 (SF3) helicase motifs conserved within genomoviruses Reps (Supplementary Table S2) [37]. The mapping of raw reads from each sample (Cap1 and Cap3) to the full-length genome sequences of the capybara-associated genomoviruses (Figure 1) confirmed that these genomoviruses were only present in sample Cap1, which is from where they were originally isolated.

**Figure 2:**
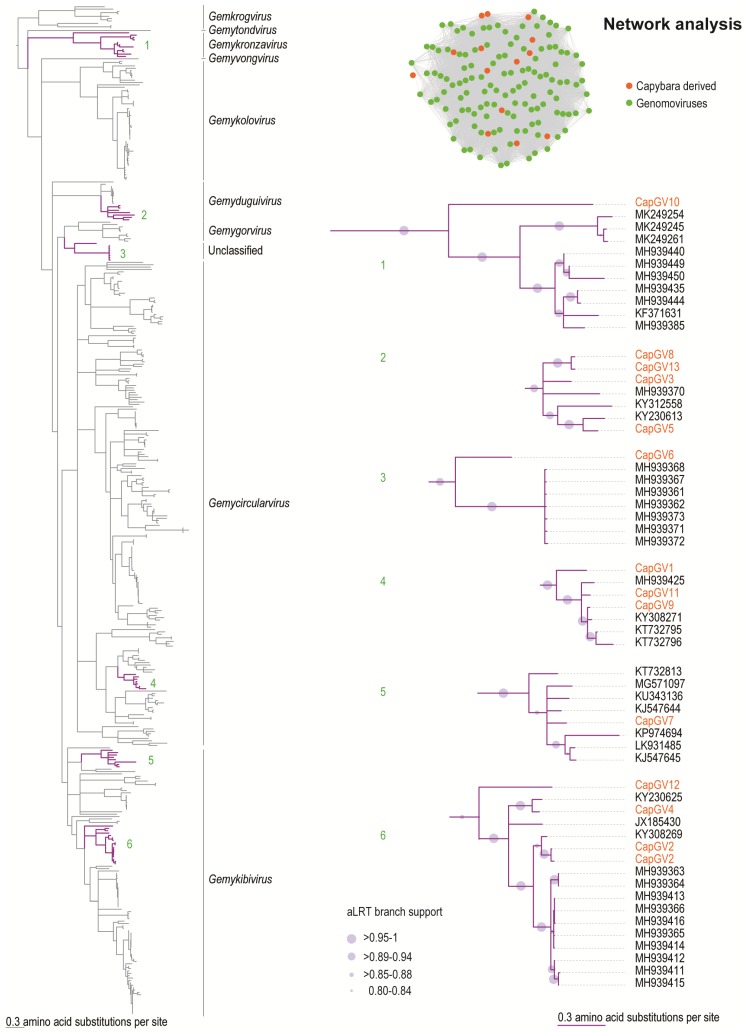
Sequence similarity network analysis and Maximum Likelihood phylogenetic tree of the Rep amino acid sequences of the viruses belonging to the nine *Genomoviridae* genera. The tree is rooted with Rep sequences of geminiviruses. Branches with aLRT support <0.8 were collapsed. The Reps of genomoviruses identified in this study are highlighted in orange. Clades containing the capybara associated genomoviruses CapGV1–13 (highlighted in purple) are expanded.

A genome-wide pairwise identity of the CapGVs with representative sequences from all known species of genomoviruses was undertaken to identify them at the species level (Supplementary Data 1). CapGV1–CapGV6, CapGV8, CapGV10, CapGV12, and CapGV13 all represent novel genomovirus species with a genome-wide identity ranging from 60% to 75% with other classified genomoviruses. Phylogenetic analysis of the Rep sequences of the genomoviruses identified in this study reveals that they can be classified within four established genera, *Gemycircularvirus* (*n* = 3), *Gemydunguivirus* (*n* = 4), *Gemykibivirus* (*n* = 5), *Gemykronzavirus* (*n* = 1), and one likely to an unclassified genus (Figure 2).

CapGV7 is a strain of sewage-associated gemycircularvirus-10a (KJ547644) [39], sharing 91% genome pairwise identity. CapGV9 is a new strain of the thrips associated genomovirus 2 (KY308271) [40], which shares 90% genome-wide identity. CapGV11 is a new strain of the plant associated genomovirus 12 (MH939425), sharing 84% genome-wide pairwise identity.

The genomovirus CapGV10 (MK483082) groups within the genus *Gemykronzavirus* (Figure 2) and has a nonanucleotide motif “TAAGATTCT.” The highest Rep and CP amino acid sequence identity is 51.3% and 34.9%, respectively, with a plant associated gemykronzavirus (MH939440) and CapGV2 (MK483073). CapGV3 (MK483075), CapGV5 (MK483077), CapGV8 (MK483080), and CapGV13 (MK483085) group with members of the genus *Gemydunguivirus* (Figure 2) and have variable nonanucleotide motifs “TAAKATTMT.” The CP and Rep amino acid sequences of CapGV3, CapGV13, and CapGV8 share 55.1%–65.3% and 85%–96% identity amongst themselves. The CP of CapG5 shares 54.1% with that of the dragonfly associated circular virus 3 (JX185428) [41], whereas its Rep shares 86.6% identity with that of the Bemisia associated genomovirus AdDF (KY230613) [42], which was identified in Brazil. 

The genomovirus CapGV6 (MK483078) has the nonanucleotide motif “TAATGTTAT” and does not group within any of the current nine established genera (Figure 2). Its Rep shares 63% amino acid identity with the plant-associated genomovirus 2 (MH939415), whereas its CP shares 72.3% sequence identity with the Pacific flying fox faeces associated gemycircularvirus 3 (KT732794) [43]. 

CapGV1 (MK483072), CapGV9 (MK483081), and CapGV11 (MK483083) group within the genus *Gemycircularvirus* (Figure 2) and have the nonanucleotide motif “TAATRTTAT.” The Rep and CP of CapGV11 share 94.7% and 78.9% amino acid sequence identity, respectively, with the plant-associated genomovirus 12 isolate LT 2029 (MH939425). The Rep and CP of CapGV9 share 97.9% and 85.9% amino acid sequence identity, respectively, with the thrips associated genomovirus 2 (KY308271) [40]. The CP of CapGV1 shares 53.3% identity with the CP of the Pacific flying fox faeces associated gemycircularvirus 5 (KT732797) [43], whereas its Rep shares 78.4% sequence identity to that of CapGV11 (MK483083) from this study.

The genomoviruses that group within the *Gemykibivirus* genus (Figure 2) are the CapGV12 (MK483084), CapGV4 (MK483076), CapGV2 (MK483073–MK483074), and CapGV7 (MK483079), which have a nonanucleotide motif of “TAATRTTAT.” The Reps of CapGV2 share 92–93% identity with that of the thrips associated genomovirus 3 (KY308269) [40], and their CPs share 53% identity with that of the Pacific flying fox faeces associated gemycircularvirus 10 (KT732805) [43]. The Rep of CapGV4 shares 93% identity with that of the Bemisia associated genomovirus (KY230625) [42], and its CP shares 69% identity with the plant-associated genomoviruses (MH939366–MH939414). The Rep and CPs of CapGV7 share 93% and 96% amino acid sequence identity with that of the sewage-associated gemycircularvirus 10a (KJ547644) [39]. The CP of CapGV12 shares 72.8% identity with the finch associated genomovirus 2 isolate E50P_A (MK249296) [44], and its Rep shares 68.3% identity with the finch associated genomovirus 2 isolate E50N_A (MK249293) [44].

#### 3.1.2. Circoviruses

The family *Circoviridae* is composed of animal-infecting ssDNA viruses with genomes of ~1.8–2.4 kb. Currently, the family is divided into two genera: *Circovirus* and *Cyclovirus*. Cycloviruses have been associated with both vertebrates and invertebrates, whereas circoviruses seem to be restricted to vertebrates [45]. 

**Figure 3:**
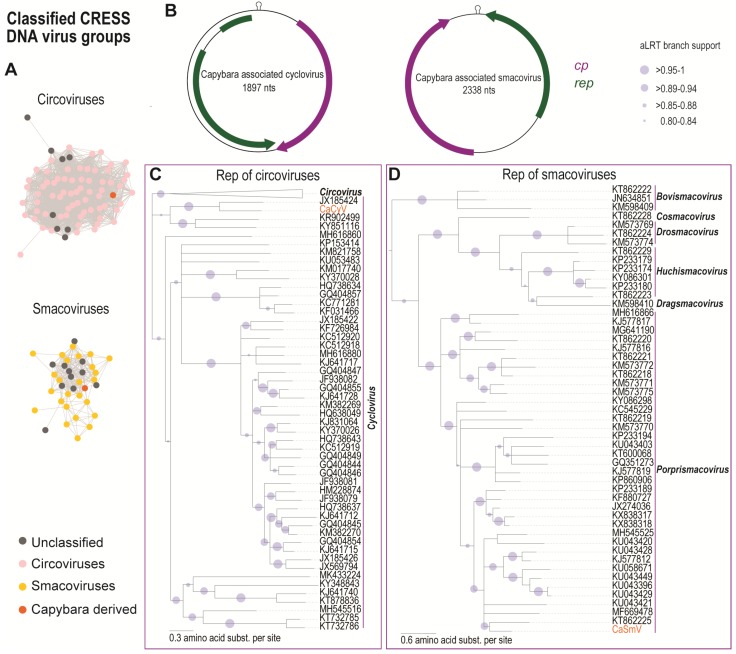
(**A**) Sequence similarity network analysis of the Rep amino acid sequences of viruses in the families *Circoviridae* and *Smacoviridae*. Orange dots represent the Rep of the viruses identified in this study. (**B**) Genome organisation of the capybara associated cyclovirus and capybara associated smacovirus. (**C**) Maximum Likelihood (ML) phylogenetic tree of the Rep amino acid sequences of the capybara associated cyclovirus with representative Rep sequences from viruses in the family *Circoviridae*. The cyclovirus Rep ML phylogenetic tree is rooted with representative sequences of unclassified CRESS DNA group (Cluster 1; Figure 4) and branches with aLRT support <0.8 have been collapsed. (**D**) ML phylogenetic tree of the Rep amino acid sequences of the capybara associated smacovirus with representative Rep sequences from viruses in the family *Smacoviridae*. The ML tree has been rooted with Reps of nanoviruses, and branches with aLRT support <0.8 were collapsed.

The Rep of the capybara associated cyclovirus (CaCyV; MK947371) identified from the capybara faeces samples clusters with those of the family *Circoviridae* (Figure 3A). The circular genome of CaCyV has 1897 nucleotides, and it encodes a replication-associated protein on the complementary sense and a capsid protein in the virion sense (Figure 3D) with the conserved nonanucleotide motif “TAGTATTAC.” Further analysis of the Rep encoded by CaCyV showed they contain the conserved RCR endonuclease and SF3 helicase motifs presented in the Rep of members of the family *Circoviridae* (Supplementary Table S2) [45]. Mapping of raw reads from each sample (Cap1 and Cap3) against the full-length genome of the CaCyV (Figure 1) revealed that the cyclovirus is common to both samples.

The Rep amino acid sequence phylogenetic analysis reveals that the new sequence belongs to the genus *Cyclovirus* (Figure 3B). CaCyV encodes a spliced Rep and represents a new species within the family sharing the highest genome-wide sequence pairwise identity of 62% (species demarcation for circoviruses is 80% genome pairwise identity [45]) with the dragonfly associated cyclovirus 5 (JX185426) [41] (Supplementary Data 2). The Rep of CaCyV shares 62.5% identity with that of the dragonfly associated cyclovirus 3 (JX185424) [41], and its CP shares 33.6% identity with that of the bat-associated cyclovirus 3 (JF938081) [46] (Supplementary Data 2). Several other cycloviruses have also been identified in animal faeces [43,46,47,48,49], suggesting that these viruses may be associated with their diet.

#### 3.1.3. Smacoviruses

Viruses in the family *Smacoviridae* have circular ssDNA genomes of ~2300–2500 nucleotides. Smacoviruses encode a Rep and CP that are bidirectionally transcribed with a conserved nonanucleotide at the origin of replication. This recently established family is classified into six genera *Bovismacovirus*, *Cosmacovirus*, *Dragsmacovirus*, *Drosmacovirus*, *Huchismacovirus,* and *Porprismacovirus* [22]. Smacoviruses have been primarily identified through the metagenomics analysis of diverse animal faecal samples. To date, these viruses have not been cultured nor has a conclusive host association been identified. A recent study provides some evidence that smacoviruses may infect archaea based on CRISPR spacers with smacovirus-like sequences identified in archaea [23].

In this study, one smacovirus (Capybara associated smacovirus, CaSmV; MK570200) was identified in the capybara faeces with a 2338 nts genome and a conserved nonanucleotide “TAGTGTTAC.” The genome encodes a Rep and a CP with two intergenic regions (Figure 3D). Mapping of raw reads from each sample (Cap1 and Cap3) to the full-length genome of the CaSmV (Figure 1) revealed that it is only present in sample Cap1, where it was initially recovered from. The Rep contains the RCR endonuclease and the SF3 helicase domain conserved within the Reps of smacoviruses [50] (Supplementary Table S2). Rep-based phylogenetic analysis reveals that CaSmV can be classified in the genus *Porprismacovirus* (Figure 3C). The CaSmV genome sequence is most closely related to the *Macaca mulatta* faeces associated virus 7 (KU043421) [51], sharing 62.7% genome pairwise identity (Supplementary Data 3). Based on the species demarcation threshold of 77% genome pairwise identity for smacoviruses [22], CaSmV is representative of a new species. The Rep of CaSmV shares 61% amino acid identity with that of the turkey associated porprismacovirus 1 (KF880727) [52], whereas the CP shares 42.3% identity with the chicken associated smacovirus (MG846353) [53].

### 3.2. Unclassified CRESS DNA Viruses

There are numerous reports of diverse CRESS DNA viruses that cannot be classified into currently established viral families. They all encode at least a Rep and a CP with different genome size and organisation (Supplementary Table S1; Supplementary Figure S2). From the two capybara faecal samples, 37 genomes were identified that cannot be classified into known families, and thus fall into the unclassified CRESS DNA virus group. These have been designated by the names capybara virus 1–37 (CapV 1–37). The CapVs range in size of 1525 to 3035 nts (Supplementary Figure S2). Out of the 37 genomes, 31 were isolated from sample Cap1, and 6 from Cap3. Mapping of raw reads from each sample (Cap1 and Cap3) to the full-length genome of the capybara unclassified CRESS DNA viruses (Figure 1) revealed that only the CapV16 genomic sequence (MK570178) that was isolated from Cap1 is common in both samples.

**Figure 4:**
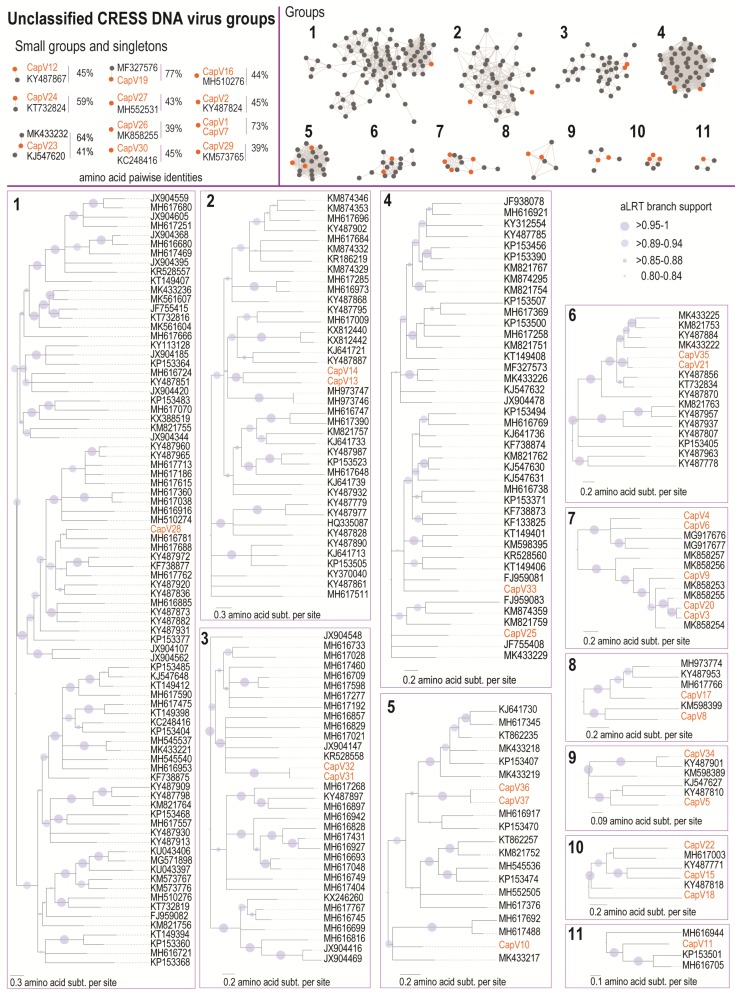
Sequence similarity network analysis and maximum likelihood phylogenetic trees of the Rep amino acid sequences of the unclassified CRESS viruses associated with the capybara. Orange dots represent the Rep of the unclassified CRESS DNA viruses identified in this study. The capybara viruses 1–37 are highlighted in orange, and branches with aLRT support <0.8 have been collapsed.

The Reps encoded by the CapVs contain the RCR endonuclease and SF3 helicase motifs that are conserved among the Reps of CRESS DNA viruses, with the exception of CapV24, which is missing the N-terminal RCR motifs I and motif II (Supplementary Table S2). Based on the sequence similarity network analysis of the Rep amino acid sequences, 24 CapVs cluster with other Reps forming 11 groups with ≥4 sequences (Figure 4). The remaining 13 sequences cluster in smaller groups or are singletons. The Rep of CapV28 clustered in group 1 shares 58.9% amino acid sequence identity with that of the CRESS DNA virus from rainbow trout tissue (MH617762) [54] (Supplementary Data 4). In group 2, the Reps of CapV13 and CapV14 share 47% and 44% amino acid sequence, respectively, with the Rep of a CRESS DNA virus sequence from wastewater (KY487868) [55] (Supplementary Data 4). Group 3 contains Reps of CapV31 and CapV32 that share 99.6% Rep amino acid identity among themselves and 48.5% with that of a Fiddler Crab associated circular virus (KR528558) [56] (Supplementary Data 4). In group 4, the Reps of CapV33 and CapV25 share 55–58% amino acid identity with that of the sewage-associated circular DNA virus 7 (KJ547631) [39] (Supplementary Data 4). In group 5, CapV10, CapV36, and Cap37 all have unidirectionally organised open reading frames. The Reps of CapV36 and CapV37 share 99.6% amino acid identity amongst themselves and 62.3% identity with that of the Lake Sarah-associated circular virus 34 (KP153470) [57] (Supplementary Data 4). The Rep of CapV10 shares 65% amino acid identity with the blackfly DNA virus 3 (MK433217) [58] (Supplementary Data 4). CapV21 and CapV35 are part of group 6, and their Reps share 92.6% amino acid identity amongst themselves and 67–72.6% with that of the Pacific flying fox faeces associated circular DNA virus 15 (KT732834) [43] (Supplementary Data 4). Group 7 contains five CapVs (CapV3, CapV4, CapV6, CapV9, and CapV20), all of which contain a putative spliced Rep and bidirectionally organised ORFs. The Reps of CapV3 and CapV20 share 89% amino acid identity. The Rep of CapV9 shares 69.6% amino acid identity with that of a tortoise associated circular virus (MK858253), and the Reps of CapV6 and CapV4 share 64% identity among themselves (Supplementary Data 4).

In group 8, the Rep of CapV17 shares 59.7% identity with that of the Apis mellifera virus 5 (MH973774) [59], while the Rep of CapV8 shares 48.6% identity with that of the Odonata associated circular virus 2 (KM598399) [60] (Supplementary Data 4). The Reps of CapV5 and CapV34 cluster in group 9 and the Rep of CapV5 shares 55.9% amino acid identity with that of a Rep from a CRESS DNA virus identified in wastewater (KY487810) [55], whereas that of CapV34 shares 76% identity with that of another CRESS DNA virus sequence from wastewater (KY487901) [55] (Supplementary Data 4). In group 10, the Rep of CapV15 shares 51.5% amino acid identity with the Rep of a CRESS DNA virus identified in wastewater (KY487771) [55] (Supplementary Data 4). The Reps of CapV18 and CapV22, both in group 10, share 48.8–52.7% identity with the Rep of a CRESS identified in wastewater (KY487818) [55] (Supplementary Data 4). Finally, in group 11, the Rep of CapV11 shares 51% identity with the Lake Sarah-associated circular virus 45 (KP153501) [57] (Supplementary Data 4). The smaller CapVs groups and singletons and their closest Rep identity to other CRESS DNA virus Reps (39–77%) are shown and summarised in Figure 4.

### 3.3. Bacteriophages

#### Microviruses

The bacterial-infecting CRESS viral family *Microviridae* is divided in two subfamilies, *Gokushovirinae* and *Bullavirinae.* They have been identified in a variety of environments, including animal gut and faecal samples [61,62,63], insects [58,59], sediments [64], seawater [65,66], and freshwater [67]. The viruses in the subfamily *Bullavirinae* are known to infect *Escherichia coli* [68]. Some viruses in the subfamily *Gokushovirinae* are known to infect *Spiroplasma*, *Chlamydia*, and *Bdellovibrio* [69,70,71]. However, there is no host association for most of those identified through viral metagenomics.

The microviruses associated with capybaras likely infect their gut microbiota or are associated with their diet. Within the two capybara faecal samples from this study, 148 microvirus genomes were identified, and of these, 80 were identified in Cap1 and 68 in Cap3. Mapping of the raw reads from each sample (Cap1 and Cap3) to the full-length genome of the capybara microviruses reveals that 36 genomic sequences are common to both samples (Figure 1). The genomes of the capybara microviruses range from 4148 to 6887 nts in size, and most encode at least the major capsid protein (MCP; pfam PHA00363), minor capsid protein (pfam PHA00327), and replication initiation protein (pfam PHA00330) (Supplementary Figure S3). 

**Figure 5:**
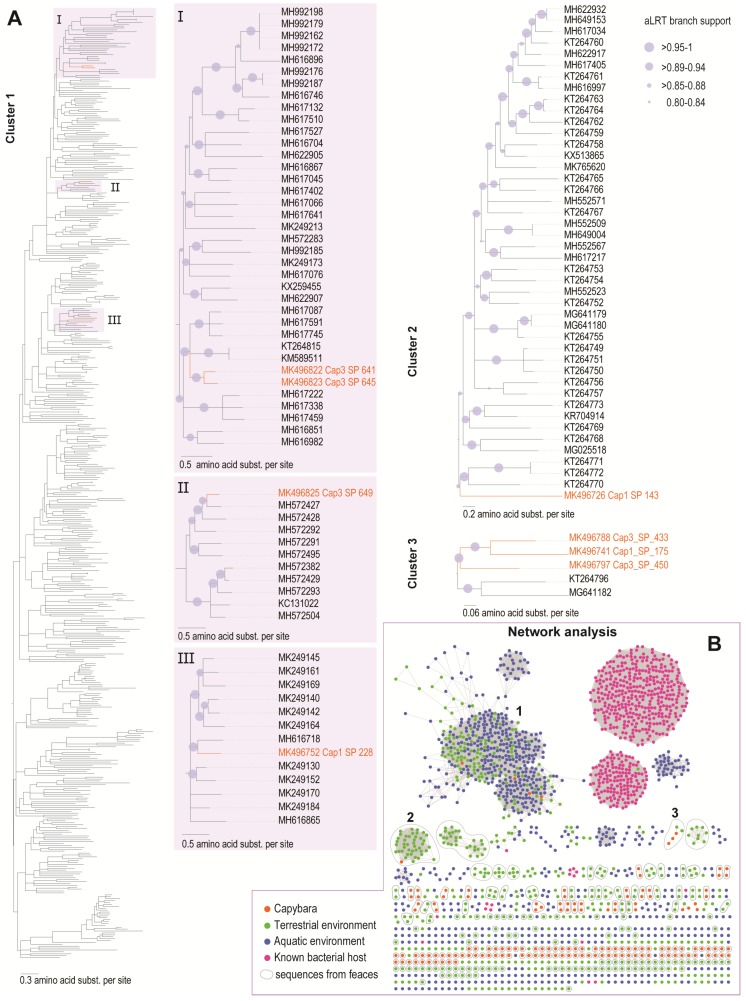
(**A**). Maximum Likelihood phylogenetic trees of the three main clusters (≥5 sequences) containing the capybara associated microviruses. The ML phylogenetic tree of cluster 1 has the branches in purple expanded (I, II, III). (**B**). Sequence similarity network of the major capsid protein (MCP) amino acid sequences of the capybara associated microviruses with a representative dataset of microviruses (MCP_all). Sequences are color-coded based on the type of environment they were recovered (terrestrial in green, aquatic in blue, and from known bacterial hosts in pink). The capybara samples are represented by orange dots, and all sequences derived from faeces are circled in grey.

The cluster analysis of the MCP, the most conserved protein of microviruses, reveals that the 148 microviruses identified in this study belong to the subfamily *Gokushovirinae*. The capybara microviruses are highly diverse with the majority (*n* = 140) of them forming groups ≤3 or as singletons (Figure 5A). Nonetheless, some sequences cluster with MCPs of microviruses identified in faecal samples (see Figure 5A). We identified three main clusters that have the capybara associated microviruses MCPs. Cluster 1 is composed of MCPs of microviruses identified in terrestrial vertebrates, with the majority of them identified in mammals. Cluster 2 and 3 MCPs are composed of those microviruses identified in faeces of mammals. 

The high diversity of the capybara associated microviruses was further confirmed with a pairwise amino acid comparison of their encoded MCP (Supplementary Data 5). The MCPs of the capybara associated microviruses share 16% to 98% amino acid sequence identity amongst themselves. From the 148 capybara associated microviruses, the most closely related at the MCP level are the isolate Cap3_SP_330 (MK496766) and isolate Cap3_SP_441 (MK496790), sharing 98% amino acid identity. The two most diverse sequences at the MCP level are from the isolates Cap3_SP_410 (MK496783) and Cap3_SP_433 (MK496788), sharing 16% amino acid identity. A summary of the top 5 BLASTp hits of the MCPs of the capybara associated microviruses from this study is provided in Supplementary Table S3.

## 4. Conclusions

The application of high throughput sequencing technology in viral metagenomics has allowed the identification of known and novel viruses in a variety of different environments. The number of CRESS DNA viruses identified to date is attributed to viral metagenomic approaches and demonstrates their ubiquity in nature. In this study, we report CRESS DNA viruses (*n* = 201) identified in two faecal samples of capybaras from Brazil. From the full virus genomes identified, 14 belong to the family *Genomoviridae*, one new species is in the family *Smacoviridae*, one new species is from the family *Circoviridae*, and 37 are unclassified diverse CRESS viruses. In addition, 148 novel microviruses were identified. Since the genomes were recovered from faeces, in the case of the prokaryotic-infecting microviruses and smacoviruses, they are likely related to the microbiota of the capybara. The correlation with a host for most CRESS viruses identified through metagenomics approach is a limitation. Nonetheless, this study expands the knowledge of viruses associated with capybaras and is the first report of single-stranded DNA viruses associated with this animal species. Some of these viruses may be infectious to these animals or associated with their microbiota or diet.

## Supplementary Materials

The following are available online at https://www.mdpi.com/1999-4915/11/8/710/s1, **Supplementary Data 1**. Pairwise identity analysis inferred using SDT v1.2 [32] of the complete genome, Rep, and CP amino acid sequences of the 14 capybara associated genomoviruses with representative sequences of the family *Genomoviridae*. **Supplementary Data 2**. Pairwise identity analysis inferred using SDT v1.2 [32] of Rep and CP amino acid sequences of the capybara associated cyclovirus 1 with representative species of the family *Circoviridae*. **Supplementary Data 3**. Pairwise identity analysis inferred using SDT v1.2 [32] of Rep and CP amino acid of the capybara associated smacovirus with representative sequences of the family *Smacoviridae*. **Supplementary Data 4**. Pairwise identity analysis inferred using SDT v1.2 [32] of Rep amino acid of the unclassified CRESS DNA viruses recovered in this study. SDT analysis was undertaken for the 11 clusters identified in the network analysis that contained the capybara viruses 1–37 (Figure 4). **Supplementary Data 5**. Pairwise identity analysis inferred using SDT v1.2 [32] of microvirus MPC amino acid sequences from the capybara microviruses recovered in this study (n-148). **Supplementary Table S1**. Summary of the viruses isolated in this study divided by the family/group in which they are classified with their accession numbers and genome orientation. **Supplementary Table S2**. Rep RCR endonuclease (Motifs I–III) and SF3 helicase motifs (Walker A, Walker B and Motif C) of cyclovirus, genomoviruses, smacovirus and unclassified CRESS DNA viruses identified in this study. **Supplementary Table S3**. Top 5 BLASTx results for MCPs of capybara associated microviruses compared against all the MCPs of all microviruses available in GenBank. **Supplementary Figure S1**. Genome organization of the Capybara associated genomoviruses presented in this study (*n* = 14). **Supplementary Figure S2**. Genome organization of the unclassified CRESS DNA viruses named Capybara virus (1–37) presented in this study. **Supplementary Figure S3**. Genome organization of the Capybara associated microviruses presented in this study (*n* = 148).
