# Supplementary material for: Single Stranded DNA Viruses Associated with Capybara Faeces Sampled in Brazil
Source: Viruses. 2019 Aug 2;11(8):710. doi: 10.3390/v11080710 (PMC6723397; doi:10.3390/v11080710)
Supplement: Supplementary file 1 [file viruses-11-00710-s001.zip › Supplementary_Figure_3.pdf]

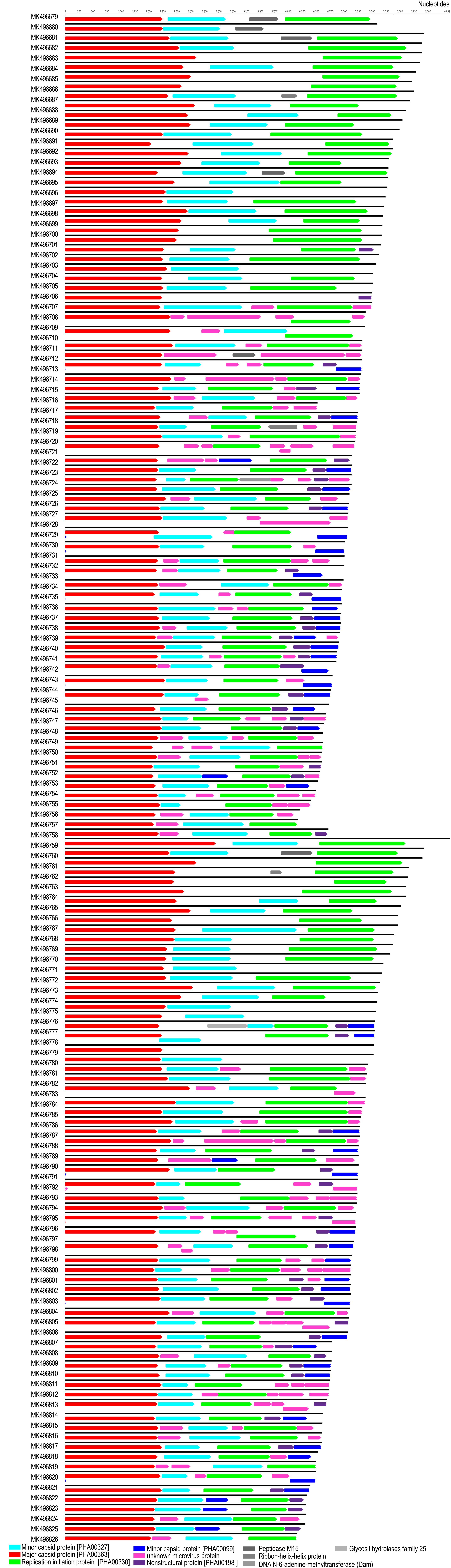
Nucleotides
MK496679
MK496680
MK496681
MK496682
MK496683
MK496684
MK496685
MK496686
MK496687
MK496688
MK496689
MK496690
MK496691
MK496692
MK496693
MK496694
MK496695
MK496696
MK496697
MK496698
MK496699
MK496700
MK496701
MK496702
MK496703
MK496704
MK496705
MK496706
MK496707
MK496708
MK496709
MK496710
MK496711
MK496712
MK496713
MK496714
MK496715
MK496716
MK496717
MK496718
MK496719
MK496720
MK496721
MK496722
MK496723
MK496724
MK496725
MK496726
MK496727
MK496728
MK496729
MK496730
MK496731
MK496732
MK496733
MK496734
MK496735
MK496736
MK496737
MK496738
MK496739
MK496740
MK496741
MK496742
MK496743
MK496744
MK496745
MK496746
MK496747
MK496748
MK496749
MK496750
MK496751
MK496752
MK496753
MK496754
MK496755
MK496756
MK496757
MK496758
MK496759
MK496760
MK496761
MK496762
MK496763
MK496764
MK496765
MK496766
MK496767
MK496768
MK496769
MK496770
MK496771
MK496772
MK496773
MK496774
MK496775
MK496776
MK496777
MK496778
MK496779
MK496780
MK496781
MK496782
MK496783
MK496784
MK496785
MK496786
MK496787
MK496788
MK496789
MK496790
MK496791
MK496792
MK496793
MK496794
MK496795
MK496796
MK496797
MK496798
MK496799
MK496800
MK496801
MK496802
MK496803
MK496804
MK496805
MK496806
MK496807
MK496808
MK496809
MK496810
MK496811
MK496812
MK496813
MK496814
MK496815
MK496816
MK496817
MK496818
MK496819
MK496820
MK496821
MK496822
MK496823
MK496824
MK496825
MK496826
Minor capsid protein [PHA00327]
Major capsid protein [PHA00363]
Replication initiation protein [PHA00330]
Minor capsid protein [PHA00099]
unknown microvirus protein
Nonstructural protein [PHA00198]
Peptidase M15
Ribbon-helix-helix protein
DNA N-6-adenine-methyltransferase (Dam)
Glycosil hydrolases family 25
