# Supplementary figures and images for: Single Stranded DNA Viruses Associated with Capybara Faeces Sampled in Brazil
Source: Viruses. 2019 Aug 2;11(8):710. doi: 10.3390/v11080710 (PMC6723397; doi:10.3390/v11080710)

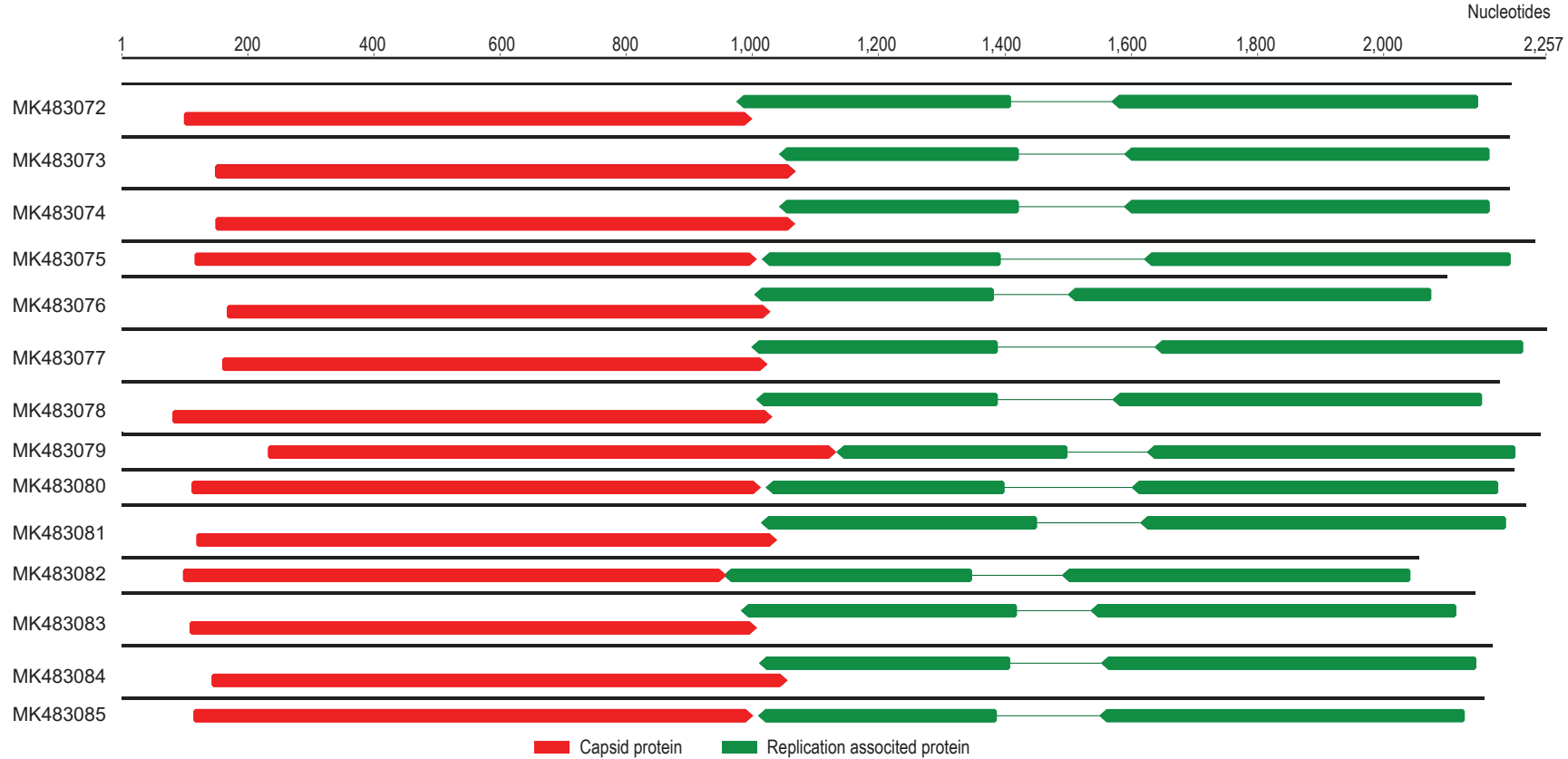

Supplement: Supplementary file 1 [file viruses-11-00710-s001.zip › Supplementary_Figure_1.pdf]

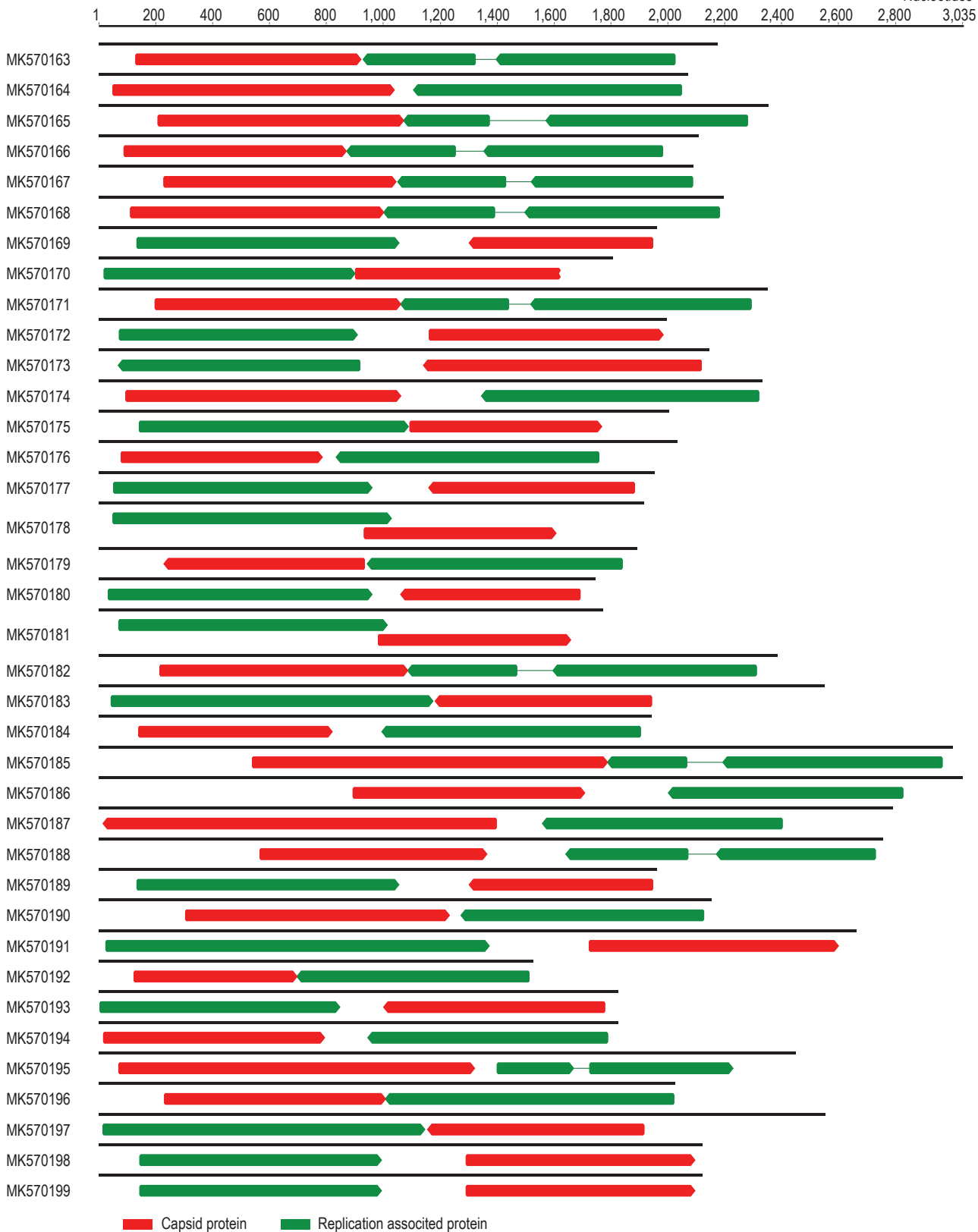

Supplement: Supplementary file 1 [file viruses-11-00710-s001.zip › Supplementary_Figure_2.pdf]
